# Whole Body Screening Using High-Temperature Superconducting MR Volume Resonators: Mice Studies

**DOI:** 10.1371/journal.pone.0033207

**Published:** 2012-04-06

**Authors:** In-Tsang Lin, Hong-Chang Yang, Jyh-Horng Chen

**Affiliations:** 1 Graduate Institute of Biomedical Electronics and Bioinformatics, National Taiwan University, Taipei, Taiwan; 2 Interdisciplinary MRI/MRS Lab, Department of Electrical Engineering, National Taiwan University, Taipei, Taiwan; 3 Department of Physics, National Taiwan University, Taipei, Taiwan; Cornell University, United States of America

## Abstract

High temperature superconducting (HTS) surface resonators have been used as a low loss RF receiver resonator for improving magnetic resonance imaging image quality. However, the application of HTS surface resonators is significantly limited by their filling factor. To maximize the filling factor, it is desirable to have the RF resonator wrapped around the sample so that more nuclear magnetic dipoles can contribute to the signal. In this study, a whole new Bi_2_Sr_2_Ca_2_Cu_2_O_3_ (Bi-2223) superconducting saddle resonator (width of 5 cm and length of 8 cm) was designed for the magnetic resonance image of a mouse's whole body in Bruker 3 T MRI system. The experiment was conducted with a professionally-made copper saddle resonator and a Bi-2223 saddle resonator to show the difference. Signal-to-noise ratio (SNR) with the HTS saddle resonator at 77 K was 2.1 and 2 folds higher than that of the copper saddle resonator at 300 K for a phantom and an in-vivo mice whole body imaging. Testing results were in accordance with predicted ones, and the difference between the predicted SNR gains and measured SNR gains were 2.4%∼2.7%. In summary, with this HTS saddle system, a mouse's whole body can be imaged in one scan and could reach a high SNR due to a 2 folds SNR gain over the professionally-made prototype of copper saddle resonator at 300 K. The use of HTS saddle resonator not only improves SNR but also enables a mouse's whole body screen in one scan.

## Introduction

Animal models have always been reasonable analogies for human research. By planting various diseases, studies can be conducted on rodents to give a closer understanding of what will happen to human. With the increase of spatial resolution, the loss of signal-to-noise ratio (SNR) in animal MR imaging needs more averaging or higher fields to compensate. Other than using high-field systems, another solution is to use high temperature superconducting materials as radiofrequency resonators [1], [2], [3], [4], [5], [6], [7], [8], [9], [10], [11], [12], [13], [14], [15], [16], [17]. Using a superconducting receiver, Hall et al. [1] imaged the back of a human head in 1991. Substantially higher SNR was demonstrated in microscopy by Black et al. [2] in 1993 using a yttrium barium copper oxide (YBCO) resonator. In the past few years, the development of relatively affordable bismuth based HTS tapes, such as Bi_2_Sr_2_Ca_2_Cu_2_O_3_ (Bi-2223) and Bi_2_Sr_2_Ca_1_Cu_2_O_3_ (Bi-2212) tapes, are gradually attracting the interest of researchers as the potential choice for RF surface resonators in MRI. Grasso et al. [7] measured the quality factors (*Q*s) of surface resonators made with Bi-2223 tapes and observed that there were only slightly less than that of YBCO films. Jing et al. [18] investigated the *Q*s theoretically and verified with experiments. Both of these investigations report that high temperature superconducting (HTS) tape resonators have the potential to obtain a higher SNR than that of copper resonators for imaging.

In the past decade, a number of investigators have shown great progress towards a superconducting surface resonator for routine MR microscopy [5], [14], [17], [19], but the practical problems of radiofrequency inhomogeneity, tuning, matching, and cryogenic temperature regulation have prevented the construction of reliable HTS volume resonators. The advantages of a HTS volume resonator are a larger field of view (FOV) and a larger filling factor than a HTS surface resonator. The design and construction of a superconducting volume resonator are considerably more complex than a traditional radiofrequency surface resonators due to the special constraints imposed by the material and the need to maintain the resonator at a low temperature [10]. In 2006, the superconducting volume resonator was demonstrated by Nouls et al. [20]. However, the superconducting volume resonator consisting of two spiral thin-film resonators in Helmholtz pair configuration was expensive and it's tuning and matching were very complex. Therefore, an easier method is proposed here for designing and fabricating industrial silver alloy sheathed Bi-2223.

In this study, for the first time to our knowledge, a Bi-2223 saddle resonator (width of 5 cm and length of 8 cm) for mice whole body images in a Bruker 3 T MRI system is demonstrated. The finite element method (FEM), which was used to construct the HTS and copper saddle resonator, has been used to design a resonator and calculate capacitance values [20], [21] in the past. The FEM is a numerical technique for finding approximate solutions of partial differential equations (PDE) as well as of integral equations. Utilizing the advantages of this saddle resonator, an attempt to compare the whole body imaging of the copper and HTS saddle resonators is done. The SNR results of the HTS saddle resonator at 77 K were compared against that of a professionally-made prototype of copper saddle resonator at 300 K for a phantom MR study to confirm the improvement. The realization of a HTS saddle resonator enables the application of HTS for a mouse's whole body imaging.

## Results

### Phantom imaging

**Figure 1:**
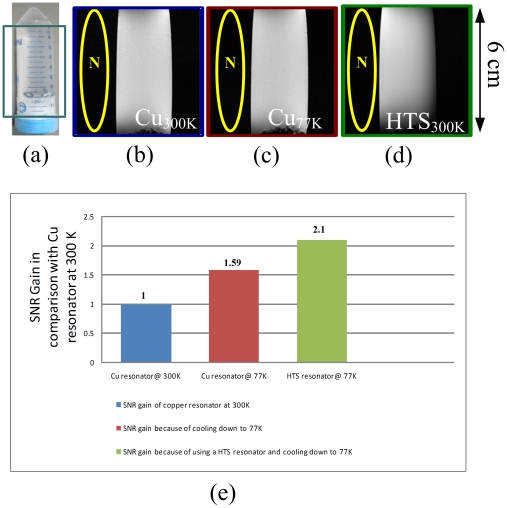
(a) A picture of the cylindrical phantom filled with 20 mM CuSO4 solution. (b) The home-made copper saddle resonator at 300 K with SNR of 115. (c) The home-made copper saddle resonator at 77 K with SNR of 182. (d) The HTS saddle resonator at 77 K with SNR of 256. (e) The comparison of SNR gain with copper and HTS saddle resonators and it clearly shows the benefit using HTS saddle resonator.

Using the phantom, the first comparison was made between the HTS and the copper saddle volume resonators. The unloaded quality factor *Q*_UL_ was 700 while the loaded quality *Q*_L_ was 518 for the HTS tape receiver resonator at 77 K. The value of *Q*_UL_ (Cu) and *Q*_L_ (Cu) for the copper receiver resonator at 300 K were 280 and 245, respectively. While the value of *Q*_UL_ and *Q*_L_ for the copper receiver resonator at 77 K were 340 and 290 at 77 K. The *Q*_L_ and *Q*_UL_ values of the HTS and the copper saddle volume resonators were bring into equation (2), the predicted SNR gain with the HTS saddle resonator at 77 K was 2.17 folds higher than that of the copper resonator at 300 K. The results were used to evaluate the performance of HTS and copper saddle resonators. The comparison of phantom images from HTS and copper saddle resonators are shown in Figure 1. Figure 1(a) is the phantom that contains 20 mM CuSO4 solution. Figure 1(b) and 1(c) represent the images acquired from the copper saddle resonator at 77 K and 300 K, respectively. Figure 1(d) represents the image acquired from the HTS surface resonator at 77 K. The SNR using the HTS tape resonator at 77 K was 256, 2.1 folds higher than that of using the copper resonator at 300 K, which was 115.

### Mice whole body imaging

**Figure 2:**
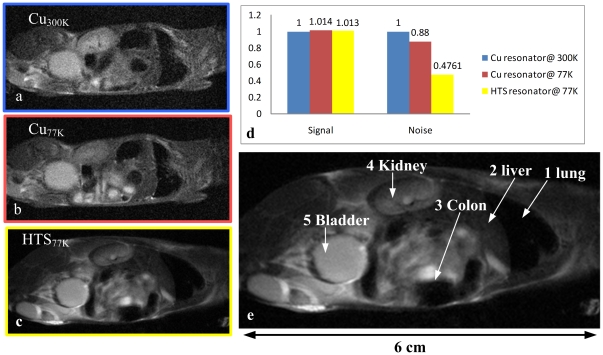
(a) The home-made copper saddle resonator at 300 K with SNR of 13. (b) The home-made copper saddle resonator at 77 K with SNR of 14.7. (c) The HTS saddle resonator at 77 K with SNR of 46. (d) The comparison of SNR gain with home-made copper and HTS saddle resonators and it clearly shows almost no increase in signal intensity and mainly from the loss of noise. (e) The mice whole body shows the anatomy illustrations of (1) lung, (2) liver, (3) intestine, (4) kidney and (5) bladder.

Further experiment was done with a mouse's whole body imaging. A HTS resonator with a 5 cm diameter and length of 8 cm was implemented to be tested for *in-vivo* mouse experiments. The comparison of a mouse's whole body images from HTS and copper saddle resonators are shown in Figure 2. Figure 2(a) and 2(b) represent the image acquired from the copper resonator at 300 K and 77 K, respectively. Figure 2(c) represents the image acquired from the HTS saddle resonator at 77 K. Since the two sets of images share the same spatial resolution, the structures and contrast are fairly the same. However, images taken with HTS saddle resonator at 77 K have less background noise. The average SNR gain from measured data of HTS saddle resonators at 77 K compared to copper saddle resonators at 300 K is 2-fold, as shown in Figure 2(d) which is mainly contributed from the noise reduction, while there is almost no increase in signal intensity. Finally, Figure 2(e) shows the anatomy illustrations of Figure 2 (c). The organs labeled in Figure 2 (e) are as follow: (1) lung (2) liver (3) intestine (4) kidney and (5) bladder.

## Discussion

### Phantom imaging

Testing results were in accordance with predicted ones, and the difference between the predicted SNR gains and measured SNR gains was 2.4% to 2.7%. Figure 1(e) shows that the increments of SNR gain from the copper resonator at 77 K and from the HTS resonator at 77 K was because they had been cooled down to 77 K. The benefit of cooling down a superconductor to 77 K and increasing SNR by 2.1-fold at the same scanning time was clearly demonstrated in Figure 1(e).

### Mice whole body imaging

As a result of the HTS saddle resonator having a 50 mm width and 80 mm length, the image is clear from the lung to the urethra. In medical diagnosis, it is quite helpful to acquire a high resolution image in a short scanning time. Although the saddle resonator can cover a larger FOV than surface resonator, its SNR is intrinsically lower than that of a surface resonator. The SNR gain using a HTS saddle resonator at 77 K was lower to 2 folds for a bigger size mice body due to the increase of the sample noise. In our previous study of HTS surface resonators, the SNR gain of rat brains was 3.5 folds and that of human hand was 1.95 folds [19]. It suggests that the bigger sample led to a lower SNR gain, which was in accordance with equation (2).

In our previous studies, high-T_c_ superconducting Bi-2223 tape surface resonators of 7 and 4 cm diameter were studied [14]. A SNR gain of 1.5-fold for kiwi fruit imaging and 2-fold for rat brain imaging over the conventional copper surface resonator at 77 K were obtained. A 200 mm diameter HTS tape surface coil was also demonstrated for phantom imaging and *in-vivo* human hand imaging using a 3 T system [19]. The sample was a 10 cm in diameter spherical phantom filled with 10 mM CuSO4 solution. Further, a phantom SNR gain of 2.22 and a human hand SNR gain of 1.95 were achieved, as compared to that of home-made copper coil. By decreasing thermal noise, the SNR can be increased several folds depending on coil dimension and main field strength. In this paper, the advantages of this saddle resonator and a comparison between the mouse's whole body imaging of a copper and a HTS saddle resonator are demonstrated. Our results show that a cooled HTS resonator provides a 2.1-fold SNR gain on phantom images over that of a copper saddle resonator at 300 K. Further, a SNR with the HTS saddle resonator at 77 K is 2 folds higher than that of a copper saddle resonator for a mouse's whole body MR study. The imaging time was greatly reduced while maintaining the same image quality.

The main question addressed by this study was whether the HTS saddle resonator shows a capability to boost SNR in animal studies. We have designed and fabricated superconducting saddle resonators for the purpose of enhancing the image quality and maximizing the filling factor of a 3 T imager. Unlike surface coils, the HTS volume coil can easily cover the entire length of a mouse, making studies of large coverage easier. Our results show that a cooled HTS resonator provided a 2.1-fold SNR gain on phantom images over that of a copper saddle resonator at 300 K. Further, a SNR with a HTS saddle resonator at 77 K is 2 folds higher than that of a copper saddle resonator for a mouse's whole body MR study. In experiment settings, using a HTS volume coil and cryostat that encapsulates the whole subject is presented might mean that heat insulation is a more challenging topic. The potential benefits justify the development of practical HTS Bi-2223 saddle resonators for imaging systems despite of considerable technical difficulties and challenges involved in the usage of cryostats and birdcage resonator design. The high-temperature superconducting saddle resonator system opens a new door for animal whole body oncology screening. Encouraged by this experiment, we will continue to improve the design of HTS saddle resonator and cryostat to fully utilize its advantages in MR imaging.

## Materials and Methods

### Theory

For samples of small size, the noise of resistance from the received saddle resonator may dominate the SNR of a MR signal. Therefore, reducing the saddle resonator resistance can effectively increase the SNR. According to the derivation by Hoult and Richards [22], the relationship between SNR and saddle resonator, can be factored and formulated as following,
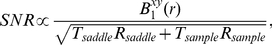
(1)Where the B_1_^xy^ represents the magnetic field produced by the unit current, T_saddle_ and T_sample_ are the temperatures of saddle resonator and sample, and R_saddle_ and R_sample_ are the resistance values of saddle resonator and sample, respectively.

Assuming that an identical magnetic field magnitude is generated by HTS and copper saddle resonators, the SNR gain of HTS saddle resonator over a copper saddle resonator is derived as following,
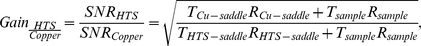
(2)


In the equation (2), Gain means SNR of the HTS saddle resonator over SNR of the copper resonator. The *T*_sample_ and *R*_sample_ both maintain the same in the experiment. Because the *R*_HTS-saddle_ and *T*_HTS-saddle_ are both smaller than those of copper resonator in 300 K. Theoretically, using the HTS saddle resonator could provide better SNR than the copper resonator in the same experiment configuration.

### Hardware

MR experiments were performed on a Bruker 3 T BioSpec MRI system (Bruker Biospin, Ettlingen, Germany) with an inserted gradient, of which the maximum gradient strength was 200 mT/m and had an inner diameter of 12 cm.

**Figure 3:**
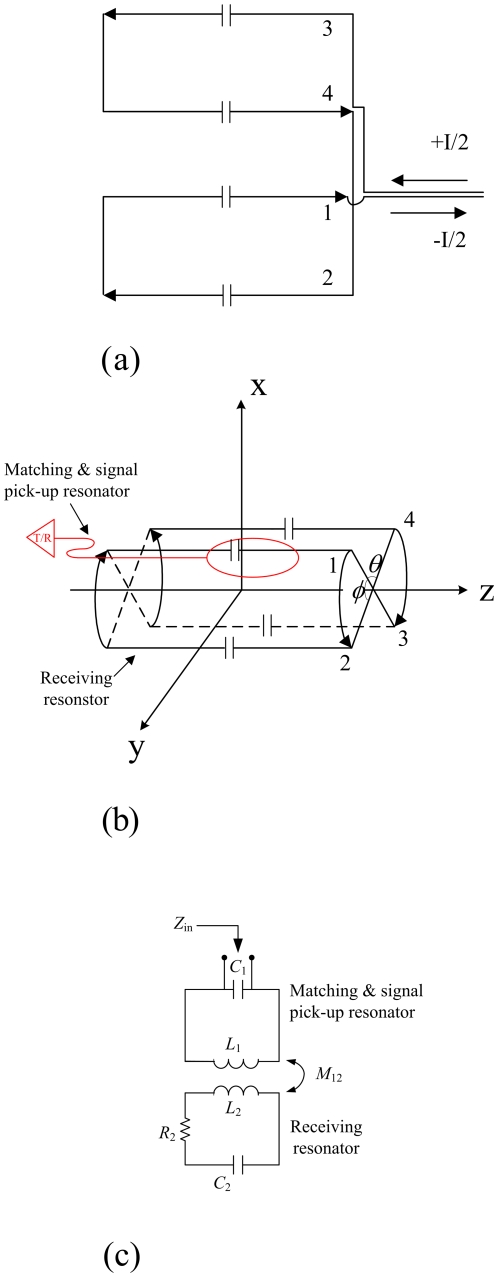
(a) The saddle shape was shown in plane from. (b) Receiving saddle resonator and pick-up resonator are indicated by the bottom one and the above one. (c) The equivalent circuit of the inductive coupled designed shows that RF signal is picked up by using the mutual inductive coupling.

The non-magnetic capacitors (56 pF, American Technical Ceramics, US) with high Q (>1000) were used in our study. The saddle resonator among the types of volume resonators is simple and easy to fabricate. For this reason, the type of saddle resonator is suitable for the comparison of the HTS and copper volume resonators. The saddle resonator is a crude approximation of the ideal current density, but it is widely used as RF resonators [23]. Two identical current wires are located on each half shell. The longitudinal wires are connected in series and driven by the same source of current I/2. Hence, the connections are made as shown in Figure 3(a). Each of the four capacitors was soldered to the middle part of the saddle resonator's legs to form a resonant circuit in Figure 3(b). The pick-up resonator was put about 3 mm under the middle of the receiving resonator. And a equivalent circuit of Figure 3(b) is shown in Figure 3(c). It should be noted that the saddle resonators were fixed in the acrylic tubes, the thickness of acrylic tubes is 1 mm, which supported the shape of the saddle resonator.

The saddle resonators were designed to have the dimension of 5 cm width and 8 cm length. To reduce the resonator resistance, Bi-2223 tape (Innova Superconductor Technology Co., Ltd., Beijing, China) was employed to fabricate the RF saddle resonators. The wires with multi-filamentary structure show a critical temperature of 110 K and an engineering critical current density greater than 9000 A/cm^2^ at 77 K. The thickness and width of the raw Bi-2223 tape are 0.23 mm and 4.1 mm, respectively, with a tin alloy sheath of 10 µm thick to provide mechanical support for the HTS Bi-2223 composition [24]. High Q capacitors were soldered with the HTS tape to obtain the desirable resonant frequency.

The equivalent copper saddle resonator used for comparison with the HTS saddle resonator in this study is a copper saddle resonator. The copper saddle resonator and the HTS saddle resonator have identical size and shapes, both were placed at the same position in the cryostat for a fair comparison.

**Figure 4:**
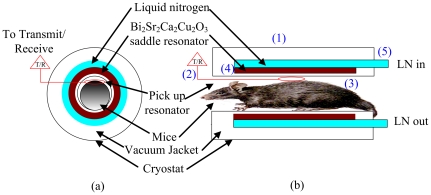
The 3 T system setup of the mice experiment, where the matching & signal pick-up coil with a tuning variable capacitance was put inside of the HTS saddle resonator. The cryostat includes the vacuum jacket and flowing liquid nitrogen.

The cryostat used for a mouse experiments was placed inside a gradient bore with a diameter of 12 cm. The separation between the HTS saddle resonator and a mouse was about 2 mm. The cryostat with a vacuum jacket was designed to provide thermal insulation. The vacuum pressure was kept below 10^−7^ torr to reduce the thermal convention. The cryostat was constructed entirely from borosilicate glass (Pyrex glass) for thermal insulation. Figure 4(a) shows the experimental setup, where the matching and signal pick-up surface resonator (20 mm in diameter) with a tuning variable capacitance were positioned between the HTS saddle resonator and the mouse. The cross sections of Figure 4(a) are also shown in Figure 4(b). Subsequently, components in Figure 4 (b) include: (1) cryostat (2) pick-up resonator (3) mouse (4) HTS saddle resonator (5) LN_2_ intake and exit. The HTS saddle resonator was cooled to the state of superconductivity by liquid nitrogen in a cryostat, which can hold a temperature of 77 K. The temperature can be maintained until the LN_2_ supply is exhausted, which can last for a time of 3 hours.

The RF signal transmission and reception were accomplished by the inductively coupled approach [25]. An inductive coupling system was designed, as shown in Figure 1(c). The inducting coupling system includes two resonators: a HTS/copper receiver saddle resonator and a pick-up surface resonator. Furthermore, one trimmer capacitor (Voltronics Corp., NJ, USA) was soldered to the pick-up surface resonator. In Figure 3(c), L_1_ is the matching coil inductance and L_2_ is the surface coil inductance, the input impedance is given by:
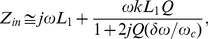
(3)where 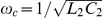 and k is the coupling constant. To create a pure resistive impedance of 50 ohm at Larmor frequency (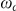), the coupling constant k must be set at an appropriate value and has to be offset from the Larmor frequency to compensate the reactance created by the inductive loop. A trimmer capacitor applied in the signal pick-up loop was tuned to the value 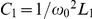 so that the imaginary part in 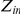 of (3) was cancelled out at the resonant frequency. Thus, pure resistive impedance 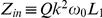 can be generated during the coil resonance. The above method was adopted to design our experiment setup. Tuning, matching and signal pick-up were done by adjusting the relative position of the signal pick-up coil and tuning a variable trimmer capacitor to cancel out the imaginary part in Z_in_. The pick-up resonator, with a diameter of 20 mm at 300 K, was approximately 2 mm away from the HTS coupled saddle resonator.

All the S-parameter measurements were performed using a network analyzer HP8751A (Agilent, CA, USA). The loaded and unloaded Q values were measured. The Q-values of both HTS and copper coupled surface resonators were measured under unloaded and loaded conditions. The Q values were calculated from the ratio of the resonance frequency at a 3 dB bandwidth.

All male BALB/c mice (age range: 8–12 weeks old; weight: 28–30 g) from the Laboratory Animal Center, National Taiwan University College of Medicine were used for experiments and were initially anesthetized with 3% isoflurane in a 1/1 Oxygen/Air mixture and injected with atropine (20 µg/kg) to avoid excessive amount of salivation. The animals were secured with a plastic head holder. The holder was warmed by a water circulation system to keep the temperature of the rat at 37°C. All the physiological data was stored in computers. The Institutional Animal Care and Use Committee at National Taiwan University approved all the procedures related to animal experiments.

### Imaging experiment

In a conventional image the SNR is measured by the mean value of pixels within the region of interest (ROI), which sizes was usually 0.5 cm by 0.5 cm. Standard deviation of background noise is measured by using the largest possible ROI (avoid ghosting/aliasing or motion artifact regions). The SNR of an image is calculated as the ratio of the mean signal to the standard deviation of the background noise. SNRs were calculated to compare the performance of HTS and copper saddle resonators. To carry out a safe test, the phantom imaging was implemented rather than *in vivo* mice imaging. The sample is a cylindrical phantom with a 29 mm diameter and length of 90 mm was filled with 20 mM CuSO_4_ solution, as shown in Figure 1(a). The optimum transmitted power was set with both HTS and copper resonators to maximize the signal amplitude. All images were obtained using the fast spin echo sequence with repetition time (TR)/echo time (TE) = 3500/62 ms and NEX = 1. The field of view (FOV), slice thickness and acquisition matrix size were 6 cm×6 cm, 1.24 mm and 256×256, respectively. The in-plane resolution was 234 µm. Total scan time was 1 min 52 s. After the phantom imaging, the imaging experiment was done on a mouse's entire body. All images were obtained using the fast spin echo sequence with TR/TE = 3500/62 ms and NEX = 1. The FOV, slice thickness and acquisition matrix size were 6 cm×6 cm, 1.24 mm and 256×256, respectively. The in-plane resolution was 234 µm. Total scan time was 7 minutes and 28 seconds.
